# Computational Thinking Training and Deep Learning Evaluation Model Construction Based on Scratch Modular Programming Course

**DOI:** 10.1155/2023/3760957

**Published:** 2023-02-22

**Authors:** Xiaoli Chen, XiaoMing Wang

**Affiliations:** ^1^Department of Educational Technology, Zhejiang Normal University, Jinhua, Zhejiang 321004, China; ^2^Key Laboratory of Intelligent Education Technology and Application of Zhejiang Province, Zhejiang Normal University, Jinhua, Zhejiang 321004, China

## Abstract

To improve the algorithmic dimension, critical thinking, and problem-solving ability of computational thinking (CT) in students' programming courses, first, a programming teaching model is constructed based on the scratch modular programming course. Secondly, the design process of the teaching model and the problem-solving model of visual programming are studied. Finally, a deep learning (DL) evaluation model is constructed, and the effectiveness of the designed teaching model is analyzed and evaluated. The T-test result of paired samples of CT is *t* = −2.08, *P* < 0.05. There are significant differences in the results of the two tests, and the designed teaching model can cause changes in students' CT abilities. The results reveal that the effectiveness of the teaching model based on scratch modular programming has been verified on the basis of experiments. The post-test values of the dimensions of algorithmic thinking, critical thinking, collaborative thinking, and problem-solving thinking are all higher than the pretest values, and there are individual differences. The *P* values are all less than 0.05, which testifies that the CT training of the designed teaching model has the algorithm dimension, critical thinking, collaborative thinking, and problem-solving ability of students' CT. The post-test values of cognitive load are all lower than the pretest values, indicating that the model has a certain positive effect on reducing cognitive load, and there is a significant difference between the pretest and post-test. In the dimension of creative thinking, the *P* value is 0.218, and there is no obvious difference in the dimensions of creativity and self-efficacy. It can be found from the DL evaluation that the average value of the DL knowledge and skills dimensions is greater than 3.5, and college students can reach a certain standard level in terms of knowledge and skills. The mean value of the process and method dimensions is about 3.1, and the mean value of the emotional attitudes and values is 2.77. The process and method, as well as emotional attitude and values, need to be strengthened. The DL level of college students is relatively low, and it is necessary to improve their DL level from the perspective of knowledge and skills, processes and methods, emotional attitudes and values. This research makes up for the shortcomings of traditional programming and design software to a certain extent. It has a certain reference value for researchers and teachers to carry out programming teaching practice.

## 1. Introduction

Since the rise of artificial intelligence (AI) as a national development strategy, AI education, programming education, and robot education have been highly valued in society, schools, and family education. Cultivating students' training thinking is an important aspect that needs attention in the programming course. Especially with the advent of the era of education informatization 2.0, all citizens are required to pay attention to the cultivation of computational thinking (CT) ability [1]. The proposition of CT conforms to the trend of the information age and provides new experiences and methods for future curriculum construction and reform. CT, Empirical Thinking, and Logical Thinking are considered to be the three major thinking models in the scientific thinking spectrum [2].

In actual programming teaching, teachers often focus on the results of problem-solving and ignore the changes in the entire thinking process of learners in solving and analyzing problems. The works or assignments created by learners become the only yardstick for judging their CT level. This kind of training model cannot improve the thinking ability of students in a real sense [3]. Scratch programming can effectively improve students' CT ability. It can be used in many aspects of teaching, such as designing animations, designing games, and solving math problems [4]. Exploring the classroom teaching implementation strategy based on scratch graphical programming in line with the current curriculum has become the top priority of information technology teaching [5]. Jiang and Li (2021) designed a scratch course aimed at improving the CT of middle school students in the teaching practice research. The result of the analysis indicated that scratch teaching improves the CT of middle school students to a certain extent [6]. Chung and Shamir (2020) studied the scratch graphical programming tool combined with the Machine Learning for Kids project to form an AI algorithm. It was applied to youth AI education for effect verification [7]. Riera et al. (2019) used the hardware logic of the perception based on the scratch teaching platform and control module as a supplement to the scratch language software logic. They also built an intelligent hardware learning system that integrates perception, processing, control, and virtual scenes [8]. The research literature suggests that CT training is mainly focused on primary and secondary schools and high schools, and there are relatively few studies in higher education. Most of the students in college have not been exposed to programming before, so the research on the cultivation of CT in college students is particularly important [9]. How to effectively cultivate CT and innovate the teaching model of CT is an important issue for college educators. The deep learning (DL) model advocates for learners to actively transfer their knowledge and apply it to solve complex problems in reality, which helps to improve learners' own critical thinking and the construction of new knowledge [10]. The learning methods are diverse, focusing on the transfer and application of knowledge, and the DL evaluation model satisfies the active and comprehensive development of learners at various aspects and levels. It transforms the actual ability of programming into the training of programming thinking, starting from scientific literacy and comprehensively inspecting students' knowledge and learning ability [11].

On account of the existing theoretical achievements, a teaching model of CT training for programming courses on the idea of scratch modular programming is proposed, which permeates the idea of modular programming in the whole process of learning. And it guides students to decompose complex problems into subproblems, realize the advantages of modular decomposition tasks, and compare CT results before and after. Moreover, the DL evaluation model is constructed to verify whether this model can effectively cultivate and train students' comprehensive analytical thinking abilities in programming teaching courses from diverse dimensions. This research has a certain positive influence on improving the algorithmic dimension, critical thinking, and problem-solving ability of students' CT and reducing cognitive load. The innovation of design lies in the combination of the scratch programming tool and CT training, and the use of the DL model for comprehensive evaluation of teaching design. Scratch programming tools can help students establish and train programming ideas, lay the foundation for learning a professional programming language, improve the transfer of students' information technology (IT) capabilities, and further make up for the deficiencies of traditional programming and design software. It has vital guiding significance for teachers and researchers to perform programming teaching practice.

## 2. Theoretical Basis and Method Research

### 2.1. The Training Elements of CT in Programming Courses

#### 2.1.1. The Concept of CT in Programming Courses

The concept of CT was first introduced in 1980 by Seymour Papert in his book, where he considered CT as a way to demonstrate the relationship between programming and thinking skills. CT can be summarized into five elements including algorithmic thinking, decomposition, abstraction, generalization, and evaluation [12]. CT applies the conceptual principles of computer science to problem-solving, models the relevant aspects of the problem, and applies the most effective methods to solve the problem. The CT process includes features such as problem structuring, data analysis, model building, algorithm design, solution implementation, and application migration [13, 14].

#### 2.1.2. The Training Elements of CT in Programming Courses

**Figure 1:**
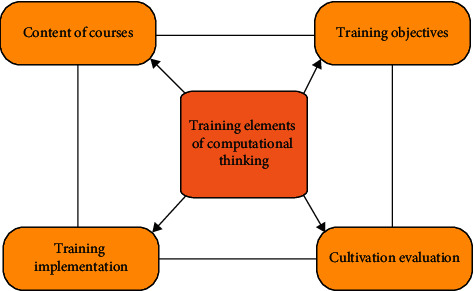
Training elements of CT.

The CT training model belongs to the subordinate concept of training mode.  Figure 1 displays the specific training elements.

The elements in Figure 1 include training objectives, content, implementation process, and evaluation. The training goal includes two aspects. One is the general goal orientation of the country, society, or school for the cultivation of students' CT, which is a macro-level goal requirement; the other is the curriculum goal of the CT training courses offered. The training content is mainly reflected in the CT syllabus, teaching content, teaching principles, teaching management methods, etc. Evaluation is the final form of testing a practical achievement.

### 2.2. Construction of a Teaching Model Based on Scratch Modular Programming

#### 2.2.1. Scratch-Based Modular Programming

Scratch is a graphical programming tool developed by the MIT Media Lab in 2007 for beginners to learn to program [15]. Scratch courses have gradually become school-based courses in some schools. Compared with other IT content, scratch teaching can stimulate students' curiosity, activate their thirst for knowledge, and help students transfer their IT capabilities. Whether it is animation or game development, it needs to abstract specific problems and stack them with instructions [16, 17]. The important performance of CT is reflected in the instruction such as selection, loop, and condition in Scratch teaching from different perspectives [18].

**Figure 2:**
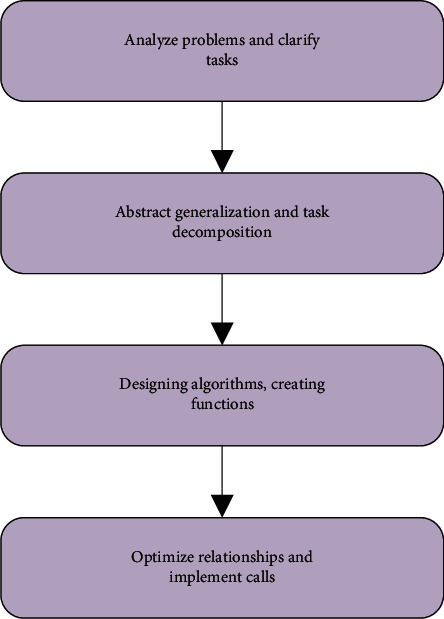
Modular programming steps.

Modular programming is a program design that divides a large program into several small program modules according to functions. Each small program module completes a certain function, and each module cooperates with each other to complete the program design of the entire function [19, 20]. The steps of modular programming are demonstrated in Figure 2.

As shown in Figure 2, the first step is to analyze the problem and clarify the tasks to be solved. The teacher guides the students to decompose and refine the task step by step and divides it into several subtasks. Each subtask only completes part of the complete function and can be implemented by functions. Next, it is necessary to determine the calling relationship between various modules, guide students to continuously debug, and optimize the calling relationship between modules. Finally, call and parallel processing are implemented in the main program.

**Figure 3:**
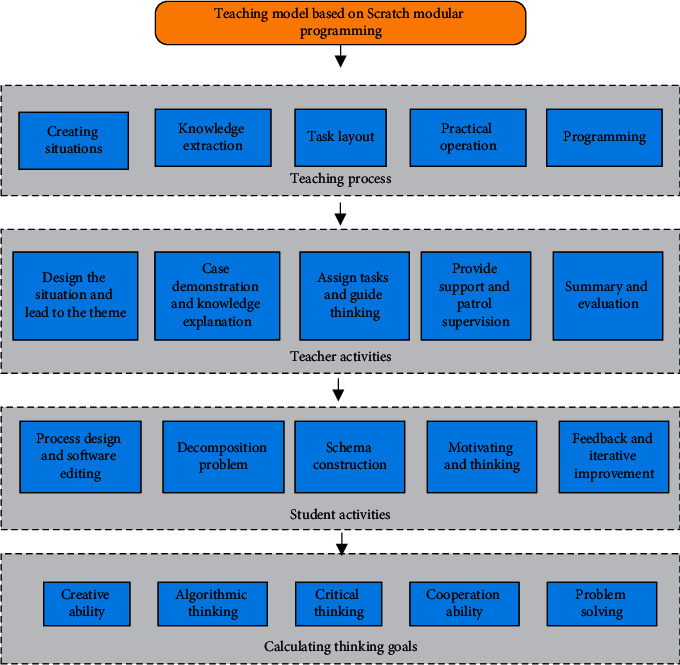
Teaching model based on the Scratch modular program.

Modular thinking training allows students to decompose complex problems into many small problems that are easy to solve. This type of thinking training can improve students' higher-order thinking abilities. This model is applied to the scratch classroom for teaching practice [21]. The teaching process mainly includes five basic links: creating a situation, refining knowledge, assigning tasks, practical operation, and summarizing and reflecting [22]. The structure of the designed teaching model based on the Scratch modular program is illustrated in Figure 3.

Figure 3 signifies that in the teaching activities, it mainly includes designing the situation and guiding the theme, case demonstration and knowledge explanation, assigning tasks and guiding thinking, support and inspection and supervision, and summary and evaluation. Student activities mainly involve process design and software editing, problem decomposition, schema construction, motivation and reflection, and feedback and improvement. In terms of CT goals, they mainly cover creativity, algorithmic thinking, collaboration, critical thinking, and problem-solving. In the process of teaching practice, in the first step, the teacher uses the grouping function to divide the students into two groups according to the programming level. In the second step, the teacher guides the students to analyze the problem and uses the Scratch modular idea to demonstrate the operation to the students. In this process, students closely link old and new knowledge and reconstruct new cognition. In the third step, teachers ask challenging questions and students answer them. In the fourth step, teachers conduct inspections and supervision, answer questions, and students visualize the plan. Finally, under the guidance of teachers, students make summaries and conduct experimental reflections and iterative improvements at this stage.

#### 2.2.2. Visual Programming Problem-Solving Model

**Figure 4:**
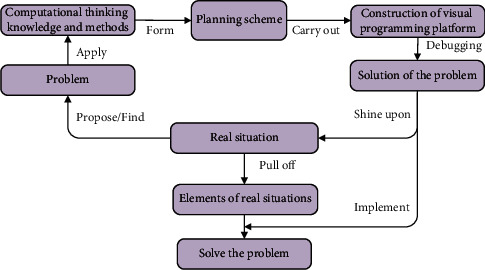
Visual programming problem-solving model.

Visual programming tools lead learners to contact the code language in the way of module splicing, which can make learners accept learning programming psychologically. Its main teaching function is to weaken the writing of programming code, emphasize the application of CT knowledge and methods, and enhance the learner's motivation [23]. Visual programming tools can describe and execute problems in real situations in a modular programming language according to the problem-solving plan [24].  Figure 4 reveals the visual programming problem-solving model.

As shown in Figure 4, a plan is formed through CT and methods, and a visual programming platform program is built according to the plan. After the platform is debugged, the solution to the problem is obtained and mapped to the real situation. Through the real situation, questions can be raised and fed back to the CT methods. The real situation can further extract the elements in the real situation, such as people, things, things and rules. Finally, the problem is solved.

### 2.3. Construction of DL Evaluation Model for CT Training

#### 2.3.1. DL Concept Theory

**Figure 5:**
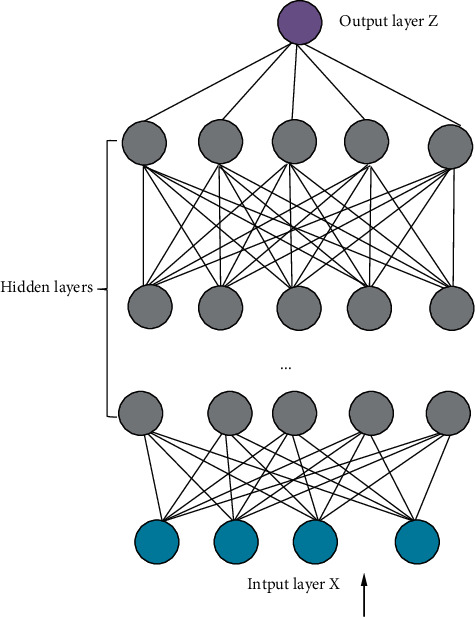
DL neural network model.

DL, also known as Deep Structure Learning, is the inherent law of learning sample data to automatically learn data features and complete tasks such as classification and regression. It has the ability to analyze, learn independently, and recognize data, such as text and images.  Figure 5 presents the DL neural network model. From bottom to top are the input layer, hidden layer, and output layer. Features are extracted layer by layer according to the feature distribution of the underlying data [25, 26].

The DL neural network model in Figure 5 uses unsupervised learning from the input layer to the output layer. In other words, the training starts from the input layer and goes up layer by layer. The parameters of each layer are trained layer by layer without calibration data. This training can be regarded as an unsupervised training process.

Assuming that *x*_1_ and *x*_2_ represent the sample features. The input layer receives the sample data features and then outputs the evaluation result. The input *x* can be expressed as the following equation:

(1) $$\begin{array}{c} x={\left(x_{1},x_{2},\cdots x_{i}\right)}^{T}\text{.} \end{array}$$

The following equation indicates the preactivation output *z*_*i*_^[*l*]^.

(2) $$\begin{array}{c} z_{i}^{\left[l\right]}=w_{i}^{{\left[l\right]}^{T}}+b_{i}^{\left[l\right]}\text{.} \end{array}$$

The activation output of the hidden layer *a*_*i*_^[*l*]^ can be written as follows:

(3) $$\begin{array}{c} a_{i}^{\left[l\right]}=g^{\left[l\right]}\left(z_{i}^{\left[l\right]}\right)\text{.} \end{array}$$

Here the superscript [*l*] represents the number of layers in the neural network, and the subscript stands for the number of neuron nodes. By training on large-scale data, representative feature information is obtained, thereby achieving the purpose of classifying and predicting sample data [27].

#### 2.3.2. Model Evaluation Calculation Based on DL

The DL model is used to evaluate the teaching model based on Scratch modular programming, and analyze the characteristics of thinking training. In the DL network model, the feature sample data X trained by CT, and the parameters *w*^[1]^, *b*^[1]^ are input to the first hidden layer, and *z*^[1]^ is calculated, as indicated in the following equation:

(4) $$\begin{array}{c} z^{\left[1\right]}=w^{\left[1\right]T}X+b^{\left[1\right]}\text{.} \end{array}$$

When finding out *a*^[*l*]^, the parameters *w*^[2]^, *b*^[2]^, and *a*^[*l*]^ are input into the second hidden layer together to solve *z*^[2]^ for the subsequent calculation of *a*^[2]^. Propagation continues according to such rules, and the whole process is called forward propagation [28]. The calculation process is presented as the following equations:

(5) $$\begin{array}{c} a^{\left[l\right]}=g\left(z^{\left[l\right]}\right)\text{,}\\ z^{\left[2\right]}=w^{\left[2\right]T}a^{\left[l\right]}+b^{\left[2\right]}\text{,}\\ a^{\left[2\right]}=g\left(z^{\left[2\right]}\right)\text{.} \end{array}$$

By analogy, *z*^[*l*]^, *a*^[*l*]^, and $\hat{y}$ can be expressed as the following equations:

(6) $$\begin{array}{c} z^{\left[l\right]}=w^{\left[l\right]T}a^{\left[l-l\right]}+b^{\left[l\right]}\text{,}\\ a^{\left[l\right]}=g\left(z^{\left[l\right]}\right)\text{,}\\ \hat{y}=a^{\left[l\right]}\text{.} \end{array}$$

An error loss function is defined as the measurement standard to make the output feature training value $\hat{y}$ gradually approach the real value *y*. The error loss function is defined as follows:

(7) $$\begin{array}{c} L\left(\hat{y},y\right)=\frac{1}{2}{\left(\hat{y}-y\right)}^{2}\text{.} \end{array}$$

The neural network model is hoped to meet the probability under certain conditions, as shown in the following equation:

(8) $$\begin{array}{c} p\left(y\;|\;x\right)=\left\{\begin{array}{c} \hat{y},\left(y=1\right)\text{,}\\ 1-\hat{y},\left(y=0\right)\text{.} \end{array}\right. \end{array}$$

(8) can be rewritten as (9).

(9) $$\begin{array}{c} p\left(y\;|\;x\right)={\hat{y}}^{y}{\left(1-\hat{y}\right)}^{1-y}\text{.} \end{array}$$

Taking the logarithm of both sides of the above results, the simplified result is shown in the following equation:

(10) $$\begin{array}{c} Blog\;p\left(y\;|\;x\right)=ylog\;\hat{y}+\left(1-y\right)\log\;\left(1-\hat{y}\right)\text{.} \end{array}$$

The larger the value of *p*(*y* | *x*), the smaller the loss. The cross-entropy function is expressed as follows:

(11) $$\begin{array}{c} L\left(\hat{y},y\right)=-\left[ylog\hat{y}+\left(1-y\right)\log\;\left(1-\hat{y}\right)\right]\text{.} \end{array}$$

#### 2.3.3. DL Features

**Figure 6:**
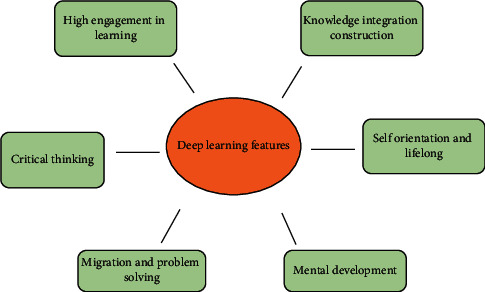
DL features.

According to the DL model and related teaching research, the basic characteristics of DL are summarized. DL is specifically summarized into six basic features [29], as expressed in Figure 6.

First, DL emphasizes a high degree of engagement in learning. Students with strong learning motivation can actively discover the meaning of knowledge, communicate and cooperate with teachers and classmates, and strive to build a knowledge system to cultivate the ability to solve practical problems. Second, DL focuses on critical understanding. Students should pay attention to understanding learning and promote DL to occur. Third, it emphasizes the integration and construction of knowledge to form a new knowledge structure. Fourth, DL attaches importance to knowledge transfer application and problem-solving. Fifth, DL pays attention to the overall development of the mind, including comprehensive practical ability, operational ability, problem-solving ability, and innovative application ability. Sixth, DL emphasizes self-direction and lifelong characteristics [30].

#### 2.3.4. DL Route

**Figure 7:**
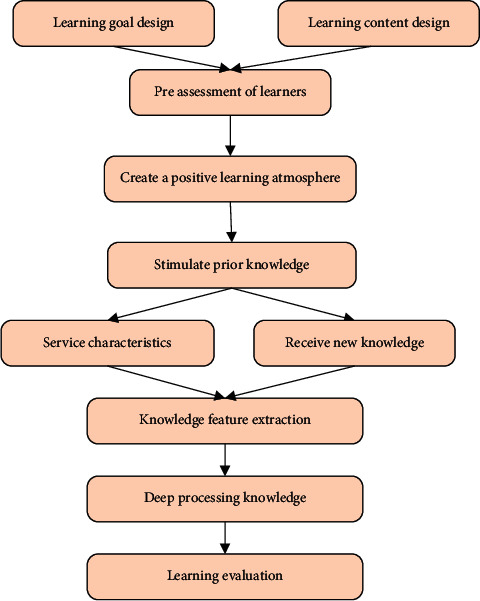
DL route.

A DL route is proposed based on DL theory, which is divided into 8 stages. First, the learning objectives and learning content are designed. Second, the learner's learning level is preassessed. Third, a positive learning atmosphere is created to stimulate previous learning. Fourth, students acquire new knowledge. Fifth, they perform feature extraction on the acquired knowledge. Sixth, they carry out in-depth processing of knowledge. Then, learners form new knowledge-cognition pairs. Finally, the learning effect is evaluated [31, 32].  Figure 7 provides the specific route.

### 2.4. Experimental Design Scheme

#### 2.4.1. Source of Experimental Data

A quasiexperiment was carried out in the scratch course of a university in Zhejiang Province to evaluate the effectiveness of the teaching model based on the idea of modular programming. The experimental subjects are 60 freshmen, all from the two classes of the 2021 educational technology major who took the Introduction to Fun Makers. The duration of the experiment was from September 2021 to December 2021, for a total of 14 teaching weeks. In this study, the test results of 10 students were randomly selected for display and comparison due to the huge data sample size, and the student numbers were defined as 1, 2, 3, 4, 5, 6, 7, 8, 9, and 10.

#### 2.4.2. Experimental Steps

**Figure 8:**
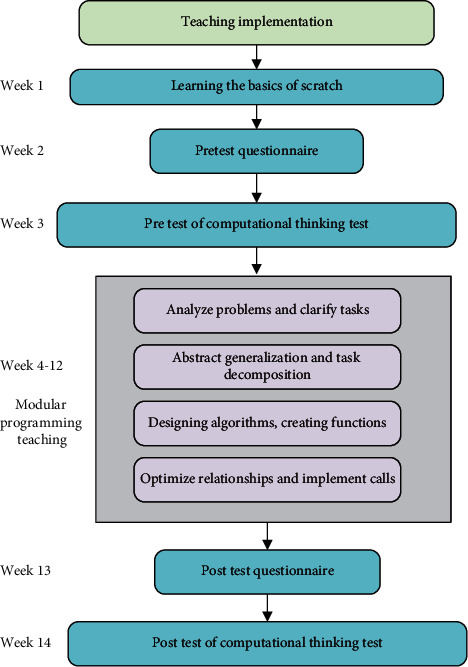
Experimental design of learning activities.

This work carried out a learning activity experiment based on the teaching model of Scratch modular programming.  Figure 8 shows the specific steps.

First, the same teacher guided the basic knowledge of Scratch programming for a week. After learning, the students were asked to complete the after-class practice task “let the kitten move”. In weeks 2 and 3, students completed the Scratch pretest questions and pretest questionnaires. The pretest questionnaire included assessments of learners' CT skills, self-efficacy, and cognitive load. In the following nine weeks, teachers taught modular programming. In the last two weeks, post-test questionnaires and post-test questions were measured and data collected. Post-test questionnaires included the Computational Thinking Scale, the Self-Efficacy Scale, and the Cognitive Load Scale. The DL model was used to evaluate the learning results.

#### 2.4.3. Measurement Tasks

The pretest and post-test questions were adapted from the Computational Thinking test (CTt), which is a tool that can assess learners' CT development level and was developed by Spanish scholar Romaán-Gonzaález. The Computational Thinking Scales (CTS) were adapted from the Likert scale, which was designed and developed by the Turkish scholar Korkmaz and others. The measurement tasks included pretest and post-test scores of CTt, pretest and post-test questionnaires of CTS, Group Self-Efficacy Scale, and Cognitive Load.

The pretest contains 10 multiple-choice questions (100%); the post-test includes 10 multiple-choice questions, each with 3 points (60%) and 10 judgment questions, each with 2 points (40%). Finally, they are converted to the percent system. The pretest and post-test questions were adapted from CTt. CTS is a five-point Likert scale consisting of 29 items. The assessment tool mainly measures the level of learners' CT ability from five dimensions: creativity, algorithmic thinking, collaboration, critical thinking, and problem-solving. The validity and reliability of the scale were studied through exploratory factor analysis, confirmatory factor analysis, item variance analysis, internal consistency coefficient, and constancy analysis. The analysis results show that the scale is an effective and reliable measurement tool for measuring students' CT ability. There are eight items in the Group Self-efficacy Questionnaire, which test individual judgments of group competence and assessments of group competence on upcoming tasks. Each item was scored on a five-point Likert scale, with 5 representing strongly agree and 1 representing strongly disagree.

Cognitive load measures include two dimensions: mental load and mental effort. The Cronbach alpha values for the two dimensions are 0.92 and 0.84, respectively. In this study, the description of the scale was adjusted to a language suitable for students' understanding according to the teacher's specific class content, and a pre- and post-test survey was conducted.

The software uses SPSS25 version and Excel to implement the data analysis.

#### 2.4.4. Test Environment for DL Evaluation

The parameter settings and operating environment of the DL evaluation model are exhibited in Table 1.

## 3. Results and Discussion

### 3.1. Paired-Sample T-Test for Student CT

SPSS25 was used to analyze the overall changes of students' CT before and after the test. The paired sample T test was used.  Table 2 lists the results.

In Table 2, the mean of the pretest was 97.45, and the mean of the post-test was 99.28, showing a rise. The standard deviation of the pretest was 13.38, and the standard deviation of the post-test was 12.42, *t* = -2.08, and *p* = 0.022 < 0.05. The results of the two tests were significantly different, indicating that the teaching model based on modular programming can cause changes in students' CT ability.

### 3.2. Multidimensional Analysis of CT Tests

#### 3.2.1. Comparison of Dimensional Tests of Creativity and Algorithmic Thinking

**Figure 9:**
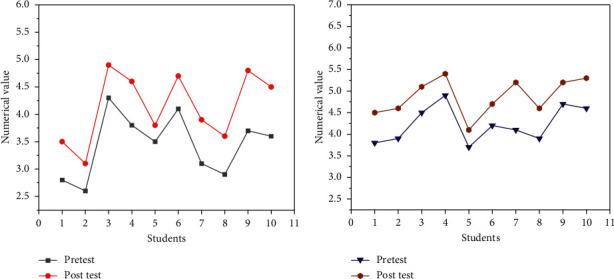
Comparison of creative thinking and algorithmic thinking in the pretest and post-test (a) comparison of creative thinking; (b) comparison of algorithmic thinking.

The CT test was conducted on 10 students from the perspective of creative thinking and algorithmic thinking. The results of the pretest and post-test were compared, as shown in Figure 9.

Figure 9 denotes that the post-test scores of the 10 students' creative thinking are higher than the pretest. Student No. 3 has the highest score. The pretest value is 4.3 the post-test score is 4.9. Student No. 2 has the lowest score, with a pretest value of 2.6 and a post-test value of 3.1. But overall, creative thinking is a growing trend. As displayed in Figure 9(b), the post-test values for the dimension of algorithmic thinking are all higher than the pretest values. Student No. 5 has the lowest score, with a pretest value of 3.7 and a post-test value of 4.1, and the overall trend is upward. The post-test values are higher than the pretest values, and creativity and algorithmic thinking abilities are improved.

#### 3.2.2. Comparison of Collaborative Thinking and Critical Thinking Tests

**Figure 10:**
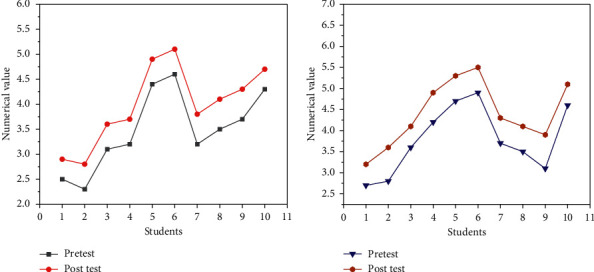
Collaborative thinking and critical thinking before and after test comparison. (a) Comparison of collaborative thinking; (b) comparison of critical thinking.

The CT was analyzed from the dimensions of Collaborative Thinking and Critical Thinking.  Figure 10 compares the results of the pretest and post-test.

In Figure 10(a), there are individual differences in the thinking of 10 students. The pretest value of student No. 2 is 2.3, and the post-test value has increased to 2.8, which is the lowest value; student No. 6 has the highest value. The post-test values of collaborative thinking in all samples are higher than in the pre-test. In the test of critical thinking in Figure 10(b), the pretest value of student No. 1 is 2.7, and the post-test value is increased to 3.2, which is the lowest value; student No. 6 is the highest. The post-test values of the critical thinking dimension are higher than the pretest values, and the overall trend also shows an upward trend.

#### 3.2.3. Comparison of Problem-Solving Ability and Self-Efficacy

**Figure 11:**
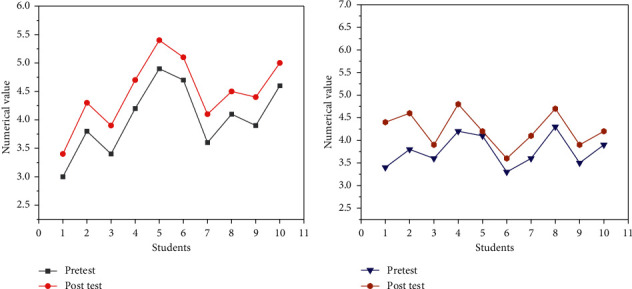
Comparison of the problem-solving ability and self-efficacy (a) comparison of problem-solving ability; (b) comparison of self-efficacy.

The CT test was performed in terms of problem-solving ability and self-efficacy.  Figure 11 presents the results of the pretest and post-test.

Figure 11(a) illustrates that the post-test value of 10 students' problem-solving ability is greater than the pretest value. Student No. 1 has the lowest value; the pretest value is 3, and the post-test value is 3.4. The overall improvement is about 0.4. The increase in the proportion is not very obvious, but to a certain extent, it can still improve the students' problem-solving ability.  Figure 11(b) refers that the post-test value of most of the students' self-efficacy is higher than the pretest, and the improvement of No. 5 student is not much, and the increase is 0.1. On the whole, students' self-efficacy has a certain improvement compared with the pretest.

#### 3.2.4. Analysis of Cognitive Load

**Figure 12:**
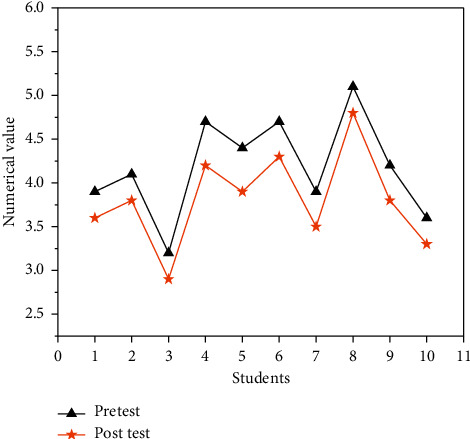
Comparison of cognitive load.

The CT test was analyzed from the perspective of cognitive load.  Figure 12 compares the results of the pre-test and post-test.

In Figure 12, the post-test values of the cognitive load of the 10 students are all lower than the pretest values, with the most obvious reductions for students No. 4 and No. 5, and the test values decreased by 0.5. Overall, the teaching model based on the idea of scratch modular programming can reduce the cognitive load of each learner to varying degrees.

### 3.3. Comparative Analysis of the Average Data of Students' CT on the Pretest and Post-test

**Figure 13:**
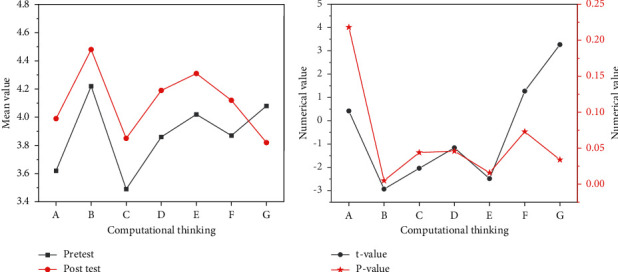
Comparison of t value and *P* value of each dimension of the pretest and post-test (a) Comparison of averages of each dimension of the pretest and post-test; (b).

A paired sample *t*-test was performed on the self-efficacy and cognitive load of CT, and the results are shown in Figure 13.

In Figure 13, A stands for creative thinking, B for algorithmic thinking, C for collaborative thinking, D for critical thinking, E for problem-solving ability, F for self-efficacy, and G for cognitive load. The post-test average of each dimension is basically higher than the pretest average of each dimension, and the post-test average of cognitive load is smaller than the pretest. The teaching model of modular programming idea has an impact on the learning effect of learners. The *P* values of algorithmic thinking, collaborative thinking, critical thinking, problem-solving ability, and cognitive load are all less than 0.05, and there are significant differences between the post-test and the pretest results. In the dimension of creative thinking, the *P* value is 0.218, and in the dimension of self-efficacy, the *P* value is 0.034. There is no obvious difference between the dimension of the two.

### 3.4. Analysis of the DL Evaluation Model

**Figure 14:**
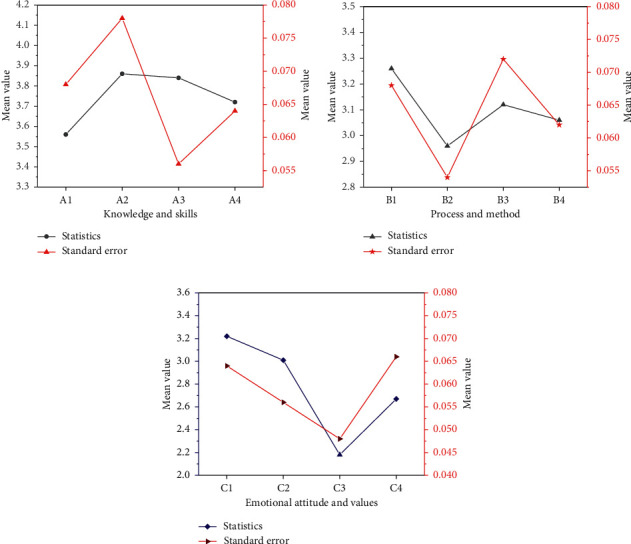
Descriptive statistics of various dimensions of DL (a) knowledge and skills dimension; (b) process and method dimension; and (c) emotional attitude and values.

The DL effect of college students were analyzed from three dimensions: knowledge and skills, process and methods, and emotional attitudes and values. SPSS25 was used to conduct overall descriptive statistics. The results are shown in Figure 14.

In Figure 14(a), A1 indicates that only knowledge points have been mastered; A2 indicates that the learned knowledge can be expressed correctly; A3 indicates that the chart and data can be explained; and A4 indicates that the learned knowledge is applied to new situations. In Figure 14(b), B1 means it is fun to learn new knowledge; B2 means that all opinions must be supported by evidence; B3 means to analyze the key points of the problem before solving the problem; and B4 means to be good at systematically planning to solve complex problems. In Figure 14(c), C1 means passing the exam with as little work as possible; C2 means that the course is uninteresting and less study time; C3 means selective learning according to necessity; and C4 means that the study irrelevant to the test is meaningless. The average value of college students' DL knowledge and skills dimensions is greater than 3.5, approaching 5, indicating that college students can reach a certain standard level in terms of knowledge and skills. The average value of the process and method dimension is about 3.1 > 3, the minimum value is 2.96, and the maximum value is 3.36, indicating that the overall level of the tested students in terms of process and method is slightly low and needs to be further improved. Finally, in terms of emotional attitudes and values, the average value is 2.77 < 3, the maximum value is 3.22, the minimum value is 2.01, the results are not satisfying. The mean values of the three dimensions are relatively concentrated, and most of them are between “disagree” and “general,” which belong to the description of uncertainty. It can be concluded that the level of DL of college students is relatively low. It is necessary to improve students' DL level from the perspective of knowledge and skills, process, method, emotion, attitude, and values.

## 4. Conclusion

To improve the CT ability of students in programming courses, a teaching model based on Scratch modular programming is implemented, and the steps of the modular programming-based teaching model and the visual programming problem-solving model are explored. To evaluate the effectiveness of the teaching model based on the idea of modular programming, a quasiexperimental method is used to conduct CT, CTS, pretest, and post-test questionnaires of the group self-efficacy scale and cognitive load. Furthermore, DL evaluation analysis is carried out on the learning results. The following conclusions are drawn. The effectiveness of the teaching model based on scratch modular programming has been verified on the basis of experiments. The T-test is performed on the paired samples of students' CT, *t* = -2.08, *P* = 0.02. There are obvious differences in the results of pretest and post-test, illustrating that the teaching model based on modular programming can cause changes in students' CT ability. The post-test values of the dimensions of algorithmic thinking, critical thinking, collaborative thinking, and problem-solving thinking are all higher than the pretest values, and there are individual differences in each sample. The *P* values are all less than 0.05, and the post-test values of cognitive load are all lower than the pretest values. It means that the CT training of the designed teaching model has a certain positive effect on the algorithm dimension, critical thinking, collaborative thinking, and problem-solving ability of students' CT and reduces cognitive load. However, there is no obvious difference in the dimensions of creativity and self-efficacy. On the basis of the DL evaluation, it can be found that college students can reach a certain standard level in terms of knowledge and skills. The process and methods, as well as emotional attitudes and values, need to be strengthened. This study also has certain limitations. Like most educational research experiments, the sample size of this experiment is not very large. There are still some limitations in the collection of learner data, and more methods and tools to effectively collect learner thinking data need to be explored. Moreover, individual differences among learners may have some influence on the experimental results. Due to the differences in individual thinking among learners, it is difficult to comprehensively consider and carry out fully targeted training in Scratch courses. The applicability of the teaching model needs to be further expanded. In the follow-up, there will be targeted training strategies and methods, and different levels of practical research will be carried out to improve the universality and effectiveness of the model many times.

## Figures and Tables

**Table 1:** Parameter settings and operating environment.

Project	Setting and model
Operating system	Ubuntu 18.04
Model of CPU	Ge force GTX 1080Ti × 2
Memory	64 GB
DL network acceleration library	cuDNN7.6.0
Programming language	Python
Network framework	PyTorch

**Table 2:** Paired sample test result.

Test	Average value	Number of cases	Standard deviation	T	Degrees of freedom	Sig (two-tailed)
Pre-CTt	97.45	60	13.38	−2.08	59	0.022
Post-CTt	99.28	60	12.42			

## Data Availability

The data used to support the findings of this study are included within the article.
